# The Effect of 10 Weeks Resistance Training on Cholesterol and Blood Triglyceride Levels of Patients with Fatty Liver Disease

**DOI:** 10.5005/jp-journals-10018-1101

**Published:** 2014-01-22

**Authors:** Rohollah Valizadeh, Siroos Hosseini Askarabadi, Sedigheh Karampour, Mona Abdolhamid Tehrani

**Affiliations:** 1 Department of Physical Education, Islamic Azad University, Omidiyeh Branch, Omidiyeh, Iran; 2 Department of Physical Education, Islamic Azad University, Behbahan Branch, Behbahan, Iran; 3 Sama Technical and Vocational Training College, Islamic Azad University, Omidiyeh Branch, Omidiyeh, Iran; 4 Sama Technical and Vocational Training College, Islamic Azad University, Omidiyeh Branch, Omidiyeh, Iran

**Keywords:** Resistance training, Cholesterol, Triglyceride, Fatty liver disease.

## Abstract

The present study aims to consider the effect of 10 weeks resistance trainings on cholesterol and blood triglyceride (TG) levels of patients with having fatty liver, aged 50 to 60 in National Iranian South Oil Company (NISOC). This research is practical and its plan has been done experimentally with pretest and post-test on experimental and control groups. In this study, 20 samples from 100 patients who referred to sonography clinic in NISOC with distinction of fatty liver were selected randomly and divided into two groups of control (n = 10) and experimental (n = 10). Cholesterol and blood trigly-ceride were measured as pretest. Test of normality for TG was (p = 0/200) by Kolmogorov-Smirnov and (p = 0/070) for cholesterol by Shapiro-Wilk test. After 10 weeks resistance trainings, the analysis and resolution of data were done by computer and SPSS (16) software as well as the descriptive and statistical methods (t-test). Comparison between these two groups showed that 8 weeks resistance trainings with a ≤ 0.05 causes significant decrease in the amount of TG but did not any significant effect on cholesterol of fatty liver patients.

**How to cite this article:** Valizadeh R, Askarabadi SH, Karampour S, Tehrani MA. The Effect of 10 Weeks Resistance Training on Cholesterol and Blood Triglyceride Levels of Patients with Fatty Liver Disease. Euroasian J Hepato-Gastroenterol 2014;4(1):64-65.

## INTRODUCTION

**Fig. 1:**
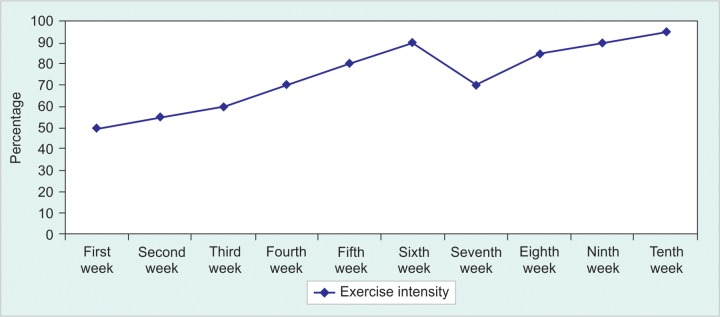
Exercise protocol

Fatty liver disease and their complications represent significant public health problem in the world.^2-4^ Patients with fatty liver diseases express increased levels of cholesterol and triglycerides (TG). These patients are advised to undertake physical exercise^1-3^ and one way to monitor prognosis of these patients may be accomplished by checking two important markers in the blood, cholesterol and TG. However, the role of physical exercises in regulating cholesterol and TG has not been fully elucidated in different parts of the world including Iran. Fatty liver diseases of these subjects were diagnosed by ultrasonography and from assessment of blood parameters. In this study, a total of 20 patients with fatty liver diseases working at National Iranian South Oil Company (NISOC), Iran, were enrolled. The ages of the patients varied from 50 to 60 years. The patients were divided in two groups: (1) patients receiving no exercise (control), and (2) the other group underwent exercise. The intensity of exercise was altered from 50 to 95% for three sessions in these patients as shown in Figure 1.

There was no significant difference in levels of cholesterol among control or experimental patients during enrollment. Also, exercise did not have any significant effect on the levels of cholesterol (Table 1).

However, the levels of TGs decreased significantly due to exercise (Table 2).

It is not clear why exercise had an effect on TGs but not on cholesterol levels. Further study with altered intensities of exercise for prolonged duration would be required as an extension of this study.

**Table 1:** **Table 1:** Comparison of cholesterol in control and experimental groups

*Stage*		*Variable*		*Groups*		*Number*		*Mean*		*Standard deviation*		*Significant level*	
Before exercise		Cholesterol		Experimental		10		192.2		31.90768		0.948	
				Control		10		193.8		29.07003			
After exercise		Cholesterol		Experimental		10		176.9		25.07965		0.167	
				Control		10		194.3		28.87540			

**Table 2:** **Table 2:** Comparison of triglyceride in control and experimental groups

*Stage*		*Variable*		*Groups*		*Number*		*Mean*		*Standard deviation*		*Degree of freedom*		*Amount of T*		*Significant level*	
Before		Triglyceride		Experimental		10		223.4		18.33758		18		-0.086		0.933	
exercise				Control		10		224.1		18.18699							
After		Triglyceride		Experimental		10		194.4		9.75477		18		-5.813		0.001	
exercise				Control		10		228		15.45603							
